# Response of Soil Bacterial Diversity, Predicted Functions and Co-Occurrence Patterns to Nanoceria and Ionic Cerium Exposure

**DOI:** 10.3390/microorganisms10101982

**Published:** 2022-10-06

**Authors:** Jie Zhang, Hui-Sheng Meng, Yan-Meng Shang, Jamie R. Lead, Zhang-Zhen Guo, Jian-Ping Hong

**Affiliations:** 1 College of Resources and Environment, Shanxi Agricultural University, Jinzhong 030801, China; 2 Center for Environmental Nanoscience and Risk, Department of Environmental Health Sciences, Arnold School of Public Health, University of South Carolina, Columbia, SC 29208, USA

**Keywords:** nanoceria, ionic cerium, bacterial diversity, co-occurrence pattern, potting soil

## Abstract

Release of nanoceria (nCeO_2_) into the environment has caused much concern about its potential toxicity, which still remains poorly understood for soil microorganisms. In this study, nanoceria and cerium (III) nitrate at different doses (10, 100 and 500 mg/kg) were applied to bok choy (*Brassica rapa* subsp. *chinensis*), grown in potting soil, to investigate the responses of soil bacterial communities to nanoceria (NC) and ionic cerium (IC) applications. The results showed that bacterial richness was slightly increased in all cerium treatments relative to the negative control without cerium amendment (CK), but a significant increase was only found in IC500. The patterns of bacterial community composition, predicted functions and phenotypes of all NC treatments were significantly differentiated from IC and CK treatments, which was correlated with the contents of cerium, available potassium and phosphorus in soil. The co-occurrence network of bacterial taxa was more complex after exposure to ionic cerium than to nanoceria. The keystone taxa of the two networks were entirely different. Predicted functions analysis found that anaerobic and Gram-negative bacteria were enriched under nanoceria exposure. Our study implies that Proteobacteria and nitrifying bacteria were significantly enriched after exposure to nanoceria and could be potential biomarkers of soil environmental perturbation from nanoceria exposure.

## 1. Introduction

Nanoceria (nCeO_2_) has broad and increasing applications in different fields, for example, as a diesel fuel additive and an industrial catalyst [1,2]. It can also be used in gas sensors in electronics [3], for corrosion protection [4] and environmental applications and as an anti-inflammatory in the biomedical area [5]. The global market volume of nanoceria was estimated to be 9100 tons in 2016 and is expected to continue to increase over time [6]. Due to its production, nanoceria has been released into the natural environment and presents potential hazards and risks [7,8].

Nanoceria as well as other engineered nanomaterials have been directly or indirectly released from the technosphere to the ecosphere and transported within environmental compartments [9]. Gottschalk et al. [10] predicted with a flow model that soils were the sink of 86.9% of the nanoceria released into the environment because of the application of sludge in the Danish environment, and the main source is direct release from production/manufacturing/use [10]. This makes it more urgent to elucidate the environmental effects of these released nanoceria in soil, which is one of the most complex niches for diverse biological communities on earth. Among the soil biota, microflora play a key role in biogeochemical processes and nutrients’ cycling [11] and are a sensitive indicator of soil quality and perturbations such as released engineered nanomaterials [12]. Previous studies have made efforts to investigate the biocompatibility and microbial toxicity of nanoceria in soils. It was found that nanoceria could alter the bacterial community structure, increase the soil microbial metabolic quotient and inhibit the microbial denitrification process [13,14,15,16]. However, knowledge on the effects of nanoceria on soil microorganisms is still limited and ambiguous, as promotive and adverse effects were both reported previously [13,16,17], and similar ambiguities exist for effects on different plant species [18,19]. Therefore, more investigation is needed to understand the effects of nanoceria on soil microorganisms.

The present study aimed to reveal the novel environmental effects of nanoceria and ionic cerium on soil bacterial diversity, community structure and co-occurrence patterns, namely: (1) the changes in soil bacterial community diversity, predicted functions and phenotypes; (2) the variations in bacterial community composition and structure as well as correlated environmental factors; (3) the differences in bacterial co-occurrence patterns after exposure and (4) the bacterial biomarkers that are significantly enriched after exposure. Our study hopes to provide a basis for better understanding the ecological effects of nanoceria and ionic cerium.

## 2. Materials and Methods

### 2.1. Experimental Design

The original soil used as the potting medium was collected from a local topsoil without any known cerium pollution (GPS coordinates: 112°34′52.83″ E, 37°25′46.82″ N). It is a calcareous cinnamon soil (Calciustepts) characterized by a moisture of 15.07%, pH of 7.6, soil organic carbon (SOC) of 4.98 g/kg, total nitrogen (TN) of 0.63 g/kg, total phosphorus (TP) of 0.68 g/kg, total potassium (TK) of 44.60 g/kg, alkali-hydrolysable nitrogen (AN) of 34.80 mg/kg, available phosphorus (AP) of 30.90 mg/kg and available potassium (AK) 288.80 mg/kg. Urea, monopotassium phosphate, potassium sulfate and composted chicken manure were added and homogenized into the original soil with a final ratio of 4.15, 2.44, 2.00 and 44.44 g/kg, respectively. Then, the soil was divided into three parts for further amendment. The nanoceria (NC) treatment group and the ionic cerium (IC) treatment group were amended with aqueous nanoceria (Suzhou Ugao Nanomaterials Co., China) or cerium (III) nitrate solution, respectively. The primary nanoceria dispersed in ultrapure water (0.2mg/L) was characterized using a multi-method approach [20,21]. The NPs’ physicochemical characteristics were as follows: the mean particle size was 25 ± 15 nm (Figure S1; SEM), the zeta potential was +14.6 mV (pH 5), the isoelectric point was 7.4 ± 2.2 and the specific surface area was 68 m^2^/g. Both groups had three treatments and the soils were amended with a gradient final cerium concentration of 10 (NC10 and IC10), 100 (NC100 and IC100) and 500 (NC500 and IC500) mg/kg. The soil in each pot was mixed throughout during the cerium amendment. A negative control group (CK) was also set with no cerium amendment. Then, three pots were filled with the soil for each treatment as replicates with 3 kg soil per pot. In total, 21 pots of seven treatments were prepared for bok choy planting.

### 2.2. Planting, Sampling and Soil Analyses

Bok choy (*Brassica rapa* subsp. *chinensis*) seedlings were purchased from a local seedling company. Seedlings with the same growth status were selected and transplanted into the prepared pots in a greenhouse. The potting bok choy was watered regularly (same amount and frequency among treatments) and harvested after 40 days. The soil in each pot was mixed after removing the bok choy, and soil samples were then collected and transported to the lab in an icebox. In total, 21 soil samples were obtained. Part of the fresh soil for each treatment was air-dried and sieved for soil physicochemical analyses and another part was stored at −80 °C for microbial analysis. SOM (soil organic matter) was calculated by multiplying the SOC concentration by 1.724 [22], and SOC was determined using the potassium dichromate oxidation method [23]. TN, TP and TK were measured using the Kjeldahl method, the molybdenum stibium colorimetric method and the Cornfield method, respectively [22,24]. AN, AP and AK were determined using the alkaline diffusion method, the sodium bicarbonate extraction method and the ammonium acetate extraction method, respectively [22]. Soil residual cerium concentration was determined using inductively coupled plasma mass spectrometry (ICP-MS, Perkin Elmer SCIEX).

### 2.3. Soil Bacterial 16S rRNA Gene Sequencing and Analysis

The soils stored at −80 °C were delivered to Gene Denovo Biological Technology Co. Ltd. (Guangzhou, China) for bacterial 16S rRNA gene sequencing. Microbial genomic DNA was extracted from 0.5 g soil using the PowerSoil DNA Isolation Kit (QIAGEN, Hilden, North Rhine Westphalia, Germany) following the manufacturer’s instructions. The quality and quantity of extracted DNA were checked using 1% agarose gel electrophoresis and a NanoDrop 2000 spectrophotometer (Thermo Fisher Scientific, Cleveland, OH, USA), respectively. The sequencing was conducted using the primer set 341F (CCTACGGGNGGCWGCAG) and 806R (GGACTACHVGGGTATCTAAT) targeting the V3-V4 region of the bacterial 16S rRNA gene [25] on an Illumina Hiseq2500 PE250 platform.

The raw reads obtained in FASTQ format were analyzed using the omicsmart cloud platform of Gene Denovo Biological Technology Co. Ltd. (Guangzhou, China) (http://www.omicsmart.com) (accessed on 5 September 2021). Briefly, the paired-end raw reads were assigned to each sample according to the barcode. After removal of the primers and barcodes, the sequences were filtered to obtain clean reads using FASTP (https://github.com/OpenGene/fastp) (accessed on 5 September 2021) by removing reads containing more than 10% ambiguous bases or less than 60% of bases with quality (Q-value) >20. Paired-end clean reads were subsequently merged into raw tags using FLASH (v1.2.11) with a minimum overlap of 10 bp and mismatch error rates of 2% [26], followed by denoising of raw tags using the QIIME (v1.9.1) [27] pipeline under specific filtering conditions [28]. Then, chimeras were checked and removed using the UCHIME algorithm (http://www.drive5.com/usearch/manual/uchime_algo.html) (accessed on 5 September 2021) to obtain high-quality reads. Then, high-quality reads with 97% similarity were assigned into the same OTU (operational taxonomic unit) using the UPARSE pipeline [29], followed by representative sequences’ selection of each OTU. Taxonomic classification of the representative sequences was performed using the RDP classifier (v2.2) [30] based on the SILVA database (v128, https://www.arb-silva.de/) (accessed on 5 September 2021) [31] with a confidence threshold value of 0.8. Then, the sequences were randomly subsampled down to the lowest number of sequences in any sample and non-bacterial reads were removed. Rarefaction curves generation and calculation of alpha diversity statistics, including the Sobs richness index, Shannon–Weaver diversity index, Pielou evenness index and Goods coverage index, were conducted in QIIME using the defined OTUs. The Adonis (Permanova) [32] test based on Bray–Curtis distance matrices was performed to determine whether there were significant differences in soil bacterial phylogenetic diversity among the treatments. Two-way ANOVA was performed to test the interaction effects of cerium species (nanoceria or ionic cerium) and dose on soil bacterial community composition at the phylum level. Bacterial taxa significantly correlated with Ce dose (*R* > 0.60, *p* < 0.05) for both nanoceria and ionic cerium were screened out using the Hmisc package (v4.4-0, https://CRAN.R-project.org/package=Hmisc) (accessed on 7 September 2021).

Bray–Curtis distances of bacterial composition between treatments at both the phylum and OTU level were calculated to perform hierarchical bi-clustering analysis using the pheatmap package (v1.0.12, https://CRAN.R-project.org/package=pheatmap) (accessed on 9 September 2021) in the R environment (v4.0.2). PCA (principal component analysis) and CAP (constrained analysis of principal coordinates) were used to depict the differentiation of the bacterial community structure among treatments using the vegan package (2.5-6, https://CRAN.R-project.org/package=vegan) (accessed on 10 September 2021) and prcomp function in R, respectively.

Linear discriminant analysis (LDA) effect size (LEfSe) was calculated to identify enriched bacterial taxa in soils under different cerium treatments. To discover the specific taxa enriched under nanoceria or ionic cerium exposure, the biomarkers in each NC or IC treatment were defined as those with significantly greater abundances than in CK, while the biomarkers of CK were those with significantly different abundances against all NC or IC treatments. A significance level of *p* < 0.05 and an effect size threshold of 3 were used for all the biomarkers.

Network analysis was performed to investigate the co-occurrence patterns of bacterial taxa between NC and IC treatments using igraph packages [33] in the R environment and the interactive platform Gephi [34] as previously described [35]. Briefly, the bacterial genera with a relative abundance >0.05% of NC treatments and IC treatments were used to calculate Spearman’s correlation coefficients. Co-occurrence events with a significant Spearman’s coefficient (*R* > 0.60, *p* < 0.01) were considered to be robust [36]. All the robust correlations jointly formed a correlation network, and each edge indicates a strong and significant correlation between the nodes. Network topology parameters were calculated as well. Nodes with a high betweenness centrality value, which indicates the relevance of a node as capable of holding together communicating nodes, were considered as keystone taxa [37,38].

PICRUSt2 (Phylogenetic Investigation of Communities by Reconstruction of Unobserved States) was employed to predict the potential functions of bacterial communities [39]. The BugBase algorithm was used to predict biologically interpretable phenotypic traits at the organism level, such as Gram status, oxygen requirements, biofilm formation and stress tolerance [40]. Hierarchical bi-clustering analysis among different treatments was performed based on both the predicted potential functions and the predicted phenotypic traits.

All statistical analyses, the multivariate analysis and the relevant graph plotting were conducted with R (v4.0.2), unless stated otherwise. Sequence data have been deposited into the Sequence Read Archive (SRA) database under accession number PRJNA760827.

## 3. Results

### 3.1. Soil Physicochemical Properties

The physicochemical properties in different treatments are displayed in Table 1. No significant differences were observed for either SOM or TN among different treatments (*p* > 0.05). Compared to CK, the contents of AN were significantly decreased in NC100, IC10 and IC100 (*p* < 0.05), while for AP, a significant decrease was observed in all NC and IC treatments except for IC10. Significant decreases in TK and AK were found in all treatments compared to CK. Additionally, all NC treatments and IC500 significantly increased the soil TP contents compared to CK (*p* < 0.05). The soil residual cerium concentration at harvest was significantly different among treatments (*p* < 0.05), with the highest being 135.03 mg/kg in NC500 and the lowest being 9.57 mg/kg in IC10. Compared to the initial adding concentration, the residual cerium concentration decreased by 73% and 79% in NC500 and IC500, respectively, and it decreased by 67% and 55% in NC100 and IC100, respectively. However, there were no significant differences in residual cerium concentration between NC10, IC10 and CK, as the average background values of cerium in soil in Shanxi Province were previously found to be 69.6 mg/kg [41]. Moreover, the soil residual cerium concentration positively correlated with TP (*R* = 0.48, *p* = 0.03) and negatively correlated with AP (*R* = −0.46, *p* = 0.03) and AK (*R*=−0. 64, *p* = 0.002).

### 3.2. Bacterial α-Diversity and Community Composition

A total of 3,371,276 raw reads of the bacterial 16S rRNA gene were obtained from the sequencing, and 3,197,402 high-quality reads were yielded after quality control and assembly. After random subsampling for homogenization, each sample had 92,969 high-quality reads. In total, 8874 OTUs defined by 97% sequence similarity were clustered in all samples. The rarefaction curves (Figure S1) based on the Sobs index (number of observed OTUs) and coverage index (Table S1) indicated that the bacterial community of all samples was well captured at the current sequencing depth.

The alpha diversity indexes of the different treatments are listed in Table S1. The Sobs richness index of all cerium treatments was higher than that of CK, but significantly higher values were only observed in IC500 compared to CK (*p* < 0.05). No significant differences were observed for the Shannon–Weaver diversity index and Pielou evenness index among the different treatments. The coverage index of all treatments was higher than 0.98, which indicated that the current sequencing depth is sufficient to saturate the bacterial diversity of all the soil samples.

**Figure 1:**
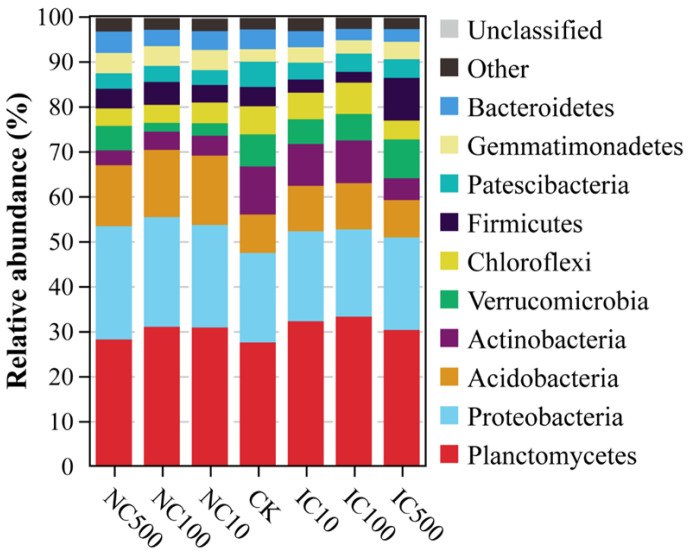
Soil bacterial community composition at the phylum level in different cerium treatments.

All the bacterial high-quality reads were classified into 35 phyla, 108 classes, 222 orders, 334 families and 661 genera, and a summary of the taxonomic classification of the 21 samples is listed in Table S2. Most high-quality reads (99.55%) could be classified at the phylum level. Planctomycetes, Proteobacteria, Acidobacteria, Actinobacteria, Verrucomicrobia, Chloroflexi, Firmicutes, Patescibacteria, Gemmatimonadetes and Bacteroidetes were the top ten dominant bacterial phyla and cumulatively accounted for more than 90% of all the taxa abundance in all treatments (Figure 1). The correlation analysis found that Proteobacteria, Lentisphaerae and Rokubacteria significantly positively correlated with nanoceria dose, while Epsilonbacteraeota had a significant negative correlation (*p* < 0.05, Table 2). For ionic cerium treatments, Hydrogenedentes and Bacteroidetes were found to positively and negatively correlate with cerium (III) nitrate dose at a significant level, respectively (*p* < 0.05).

### 3.3. Differences in Soil Bacterial Community Patterns

The two-way ANOVA revealed that both Ce species and dose had a significant effect (*p* < 0.05) on the abundance of several bacterial phyla (Table S3). However, no significant interactive effects (*p* < 0.05) of Ce species and dose were observed other than for Fibrobacteres. Among the top ten phyla, Acidobacteria, Actinobacteria, Verrucomicrobia, Chloroflexi and Gemmatimonadetes were significantly affected by both Ce species and dose (*p* < 0.05). Proteobacteria and Bacteroidetes were just under the significant one-way effect of Ce species (*p* < 0.05).

**Figure 2:**
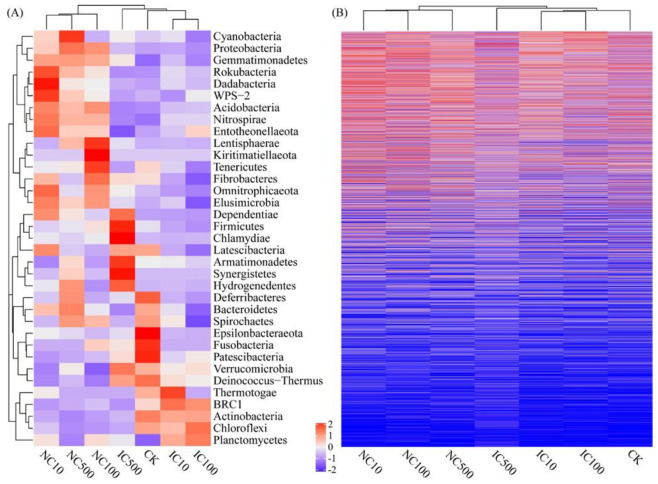
Hierarchical clustering heatmaps based on bacterial community at phylum (**A**) and OTU (**B**) level. The heatmaps are based on the normalized taxa relative abundance data. Warmer color (red) indicates a higher taxa abundance, while colder color indicates a lower taxa abundance.

To reveal the dissimilarities of bacterial community patterns after different cerium treatments, hierarchical bi-clustering analysis was performed at both the phylum and OTU level, and the corresponding heatmaps were plotted (Figure 2). It was observed that at both the phylum level (Figure 2A) and the OTU level (Figure 2B), all three NC treatments were obviously differentiated from all IC treatments and CK, which were grouped in the same cluster. Proteobacteria, Gemmatimonadetes, Acdidobacteria, Nitrospirae and Elusimicrobia showed a similar distribution pattern among the different treatments, which was consistent with the clustering pattern of the treatments (Figure 2A)—i.e., these bacterial phyla similarly had a higher abundance in the NC cluster and a lower abundance in the other cluster (IC and CK).

**Figure 3:**
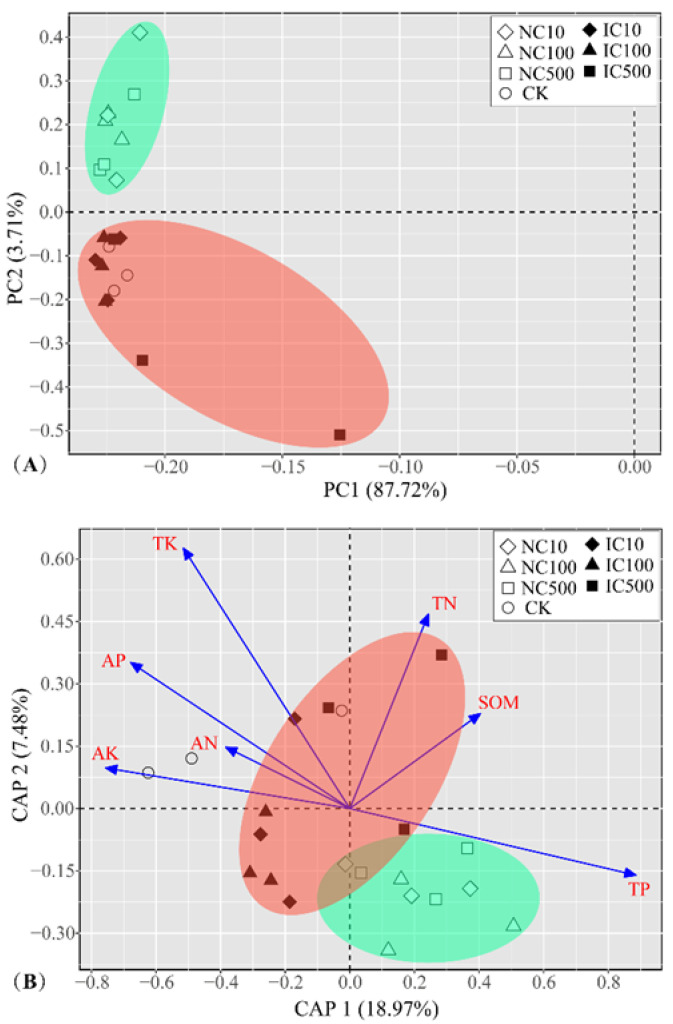
Coordinate biplot of PCA (**A**) and CAP (**B**) for soil bacterial community composition at genus level. PCA, principal component analysis; CAP, constrained analysis of principal coordinates.

A PCA was performed based on the bacterial genera composition, and the coordinate biplot is shown in Figure 3A. The first two PC axes collectively explained 91.43% of the bacterial community composition variations among the different cerium treatments. The first axis obviously separated the NC treatments from the IC treatments and CK. A CAP was also performed to further investigate the differentiation of the bacterial genera community composition among different treatments and the key shaping factors. As presented in Figure 3B, the first two axes explained 29.41% of the variation. The NC treatments were obviously separated from the IC treatments and CK, which was similar to the PCA biplot. It showed that residual Ce, TP, AP and AK were the important factors shaping the differentiation of IC and NC treatments. Furthermore, the Adonis test indicated that the bacterial genera composition differentiation among the NC and IC treatments was significant with *R*^2^ = 0.24 and *p* = 0.001. Both PCA and CAP biplots indicated that the bacterial community composition was different between the NC and IC treatment groups, but no obvious differentiation was observed between dose treatments within each group.

### 3.4. Bacterial Biomarkers under Cerium Exposure

**Figure 4:**
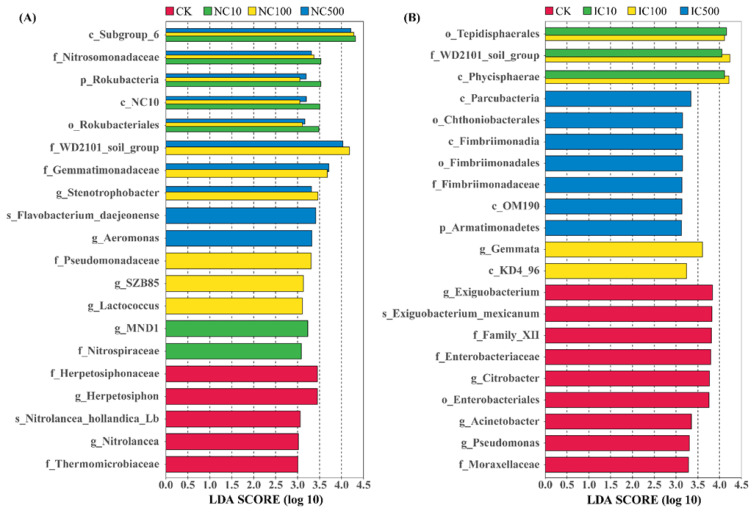
LDA scores of bacterial taxa in NC (**A**) and IC treatments (**B**). The biomarkers in each NC or IC treatment were defined as those significantly enriched only compared to CK, while the biomarkers of CK were those with significantly different abundances compared to all NC or IC treatments. A significance level of *p* < 0.05 and an effect size threshold of 3 were used for all the biomarkers. The lowercase prefix letters indicate the taxonomic level of the biomarker: p = phylum; c = class; o = order; f = family; g = genus; s = species.

The LEfSe algorithm was used to compare two or more treatments to identify the bacterial biomarkers that were significantly enriched. The taxa with an LDA score >3.0 from each treatment are depicted in Figure 4. Compared to the NC treatments, the five biomarkers enriched in CK belonged to Chloroflexi. They are Herpetosiphonaceae and *Herpetosiphon* of Chloroflexales and Thermomicrobiaceae, *Nitrolancea* and *Nitrolancea hollandica Lb* of Thermomicrobiales (Figure 4A). Compared to CK, five biomarkers were identified in all NC treatments, including Subgroup_6 of Acidobacteria, Nitrosomonadaceae of Proteobacteria, c_NC10 and Rokubacteriales of Rokubacteria and Rokubacteria itself (Figure 4A). Three biomarkers were enriched in NC100 and NC500, namely WD2101_soil_group of Planctomycetes, Gemmatimonadaceae of Gemmatimonadetes and *Stenotrophobacter* of Acidobacteria (Figure 4A). More than half of the taxa exclusively enriched in either of the NC treatments were assigned to Proteobacteria, such as Aeromonas, Pseudomonadaceae, SZB85 (Nitrosococcaceae) and MND1 (Nitrosomonadaceae).

Compared to the IC treatments, nine bacterial taxa were significantly enriched in CK, including three Bacillales taxa of Firmicutes (Family_XII, *Exiguobacterium* and *Exiguobacterium mexicanum*) and six Gammaproteobacteria taxa of Proteobacteria (Figure 4B). Two of the six Gammaproteobacteria belonged to Enterobacteriales and three belonged to Pseudomonadales (Moraxellaceae and Pseudomonadaceae). Compared to CK, three Phycisphaerae taxa of Planctomycetes (Phycisphaerae, Tepidisphaerales and WD2101_soil_group) were enriched in both IC10 and IC100. For IC500, three Armatimonadetes taxa (Fimbriimonadia, Fimbriimonadales and Fimbriimonadaceae), one Patescibacteria (Parcubacteria), one Verrucomicrobia taxon (Chthoniobacterales) and one Planctomycetes taxon (OM190) were enriched. Two taxa of Planctomycetes (Gemmata) and Chloroflexi (KD4_96) were exclusively enriched in IC100.

### 3.5. Comparison of Co-Occurrence Network Patterns of NC and IC Treatments

The bacterial co-occurrence networks of nanoceria- and ionic cerium-treated soil were generated based on the robust co-occurrence events between the predominant bacterial genera. The topological properties of the two co-occurrence networks are presented in Table 3. The two networks of NC and IC consisted of 82 and 91 nodes (genera), respectively. The number of edges, representing the robust correlations between bacterial genera, was 101 for the NC network and 214 for the IC network. The IC network had a higher average degree of 4.703 than the NC network at 2.463. The diameters of the networks were 9 (NC) and 16 (IC), while the graph density was higher in the IC network. The modularity and clustering coefficient were all found to be higher in the NC network. The modularity was 0.781 for the NC network and 0.719 for the IC network, both of which were >0.4, indicating a modular structure.

**Figure 5:**
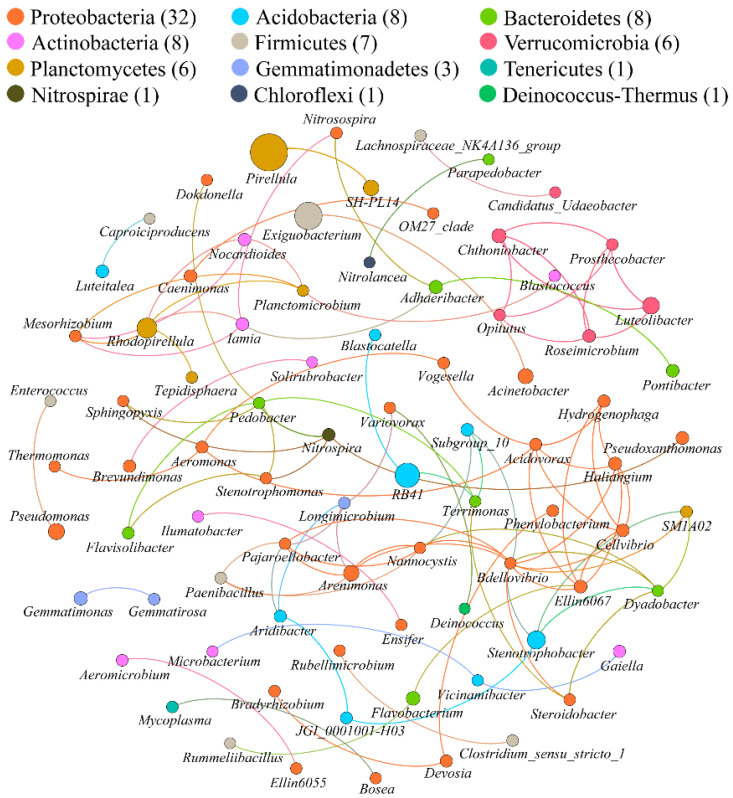
Network of co-occurring bacterial genera of nanoceria treatments based on Spearman correlation analysis sorted in color by phylum. A connection indicates a significant (*r* > 0.6, *p* < 0.01) correlation. The size of each node (genus) is proportional to its abundance; the thickness of each connection between two nodes (edge) is proportional to the corresponding correlation coefficient.

The 82 nodes (genera) in the NC network belonged to 12 phyla (Figure 5). Among them, nodes from Proteobacteria, Acidobacteria, Bacteroidetes, Actinobacteria, Firmicutes, Verrucomicrobia and Planctomycetes were predominant with a cumulative abundance of 91.5%. Proteobacteria constituted 39.02% of the nodes. All 82 nodes were clustered into 20 modules (Figure S3A), among which the first four modules constituted 54.88% of the nodes.

According to the betweenness centrality scores of each node, the top ten identified keystone genera were, in descending order of the score, as follows: *Bdellovibrio*, *Terrimonas*, *Pedobacter*, Subgroup_10, Ellin6067, *Steroidobacter*, *Acidovorax*, *Pajaroellobacter*, *Longimicrobium* and *Arenimonas*. Among the ten keystone taxa, six were assigned to Proteobacteria, while two were Bacteroidetes, one was Acidobacteria and one was Chloroflexi.

**Figure 6:**
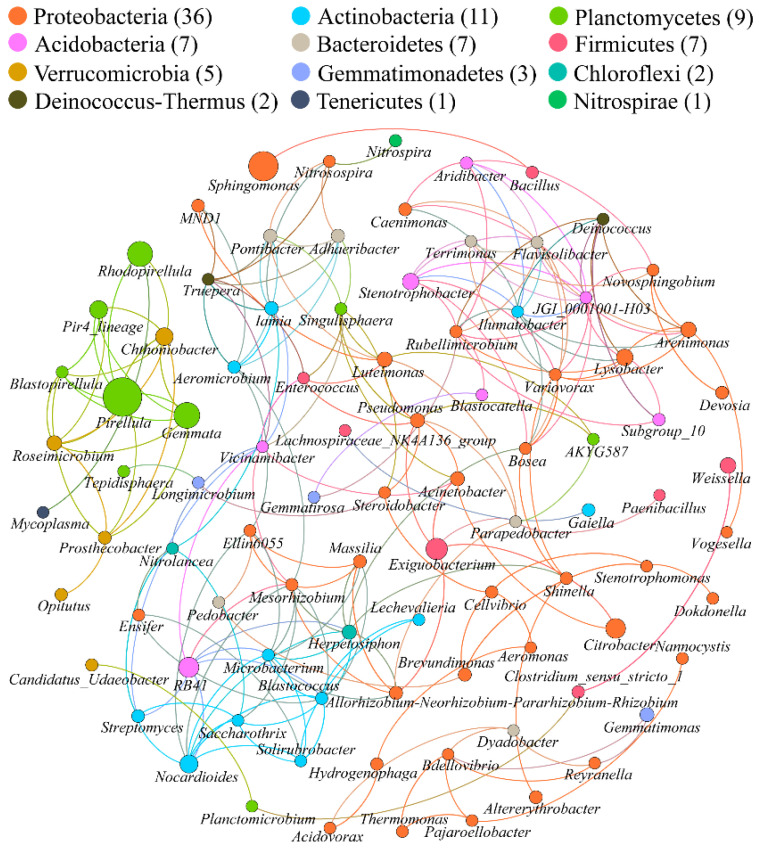
Network of co-occurring bacterial genera in ionic cerium treatments based on Spearman correlation analysis sorted in color by phylum. A connection indicates a significant (*r* > 0.6, *p* < 0.01) correlation. The size of each node (genus) is proportional to its abundance.

For the IC network, all 91 nodes (genera) were assigned into 12 phyla (Figure 6). Among them, nodes from Proteobacteria, Actinobacteria, Planctomycetes, Acidobacteria, Bacteroidetes and Firmicutes were predominant with a cumulative abundance of 84.62%. Proteobacteria, the most predominant phylum, constituted 39.56% of the nodes. All 91 nodes were clustered into nine modules (Figure S3B), among which the first four modules included 71.43% of the nodes in total. According to the betweenness centrality scores of each node, the top ten identified keystone genera were, in descending order of the score, as follows: *Herpetosiphon*, *Bosea*, *Cellvibrio*, *Steroidobacter*, *Aeromonas*, *Hydrogenophaga*, *Acidovorax*, *Mesorhizobium*, *Vicinamibacter* and *Dyadobacter*, seven of which were Proteobacteria while the other three belonged to Chloroflexi, Acidobacteria and Bacteroidetes.

### 3.6. Differences in Predicted Function and Phenotypic Traits

**Figure 7:**
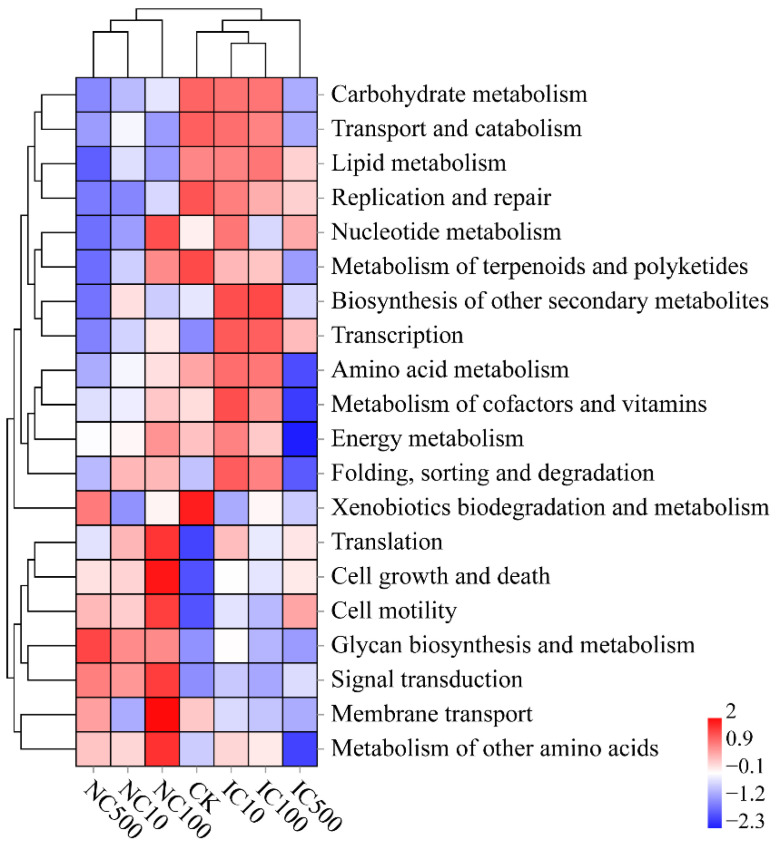
Hierarchical clustering heatmap of predicted bacterial potential functions by PICRUSt2. The heatmap is based on the normalized function abundance data. Warmer color (red) indicates a higher function abundance, while colder color indicates a lower function abundance.

The potential functions of the soil bacterial community were predicted using PICRUSt2. The hierarchical heatmap clustering analysis revealed that the function patterns of NC treatments obviously differed from those of IC treatments and CK (Figure 7). The heatmap showed that the relative abundance of lipid metabolism and replication and repair was lower in NC treatments than in IC treatments and CK, while the relative abundance of glycan biosynthesis and metabolism and signal transduction was higher in NC treatments.

**Figure 8:**
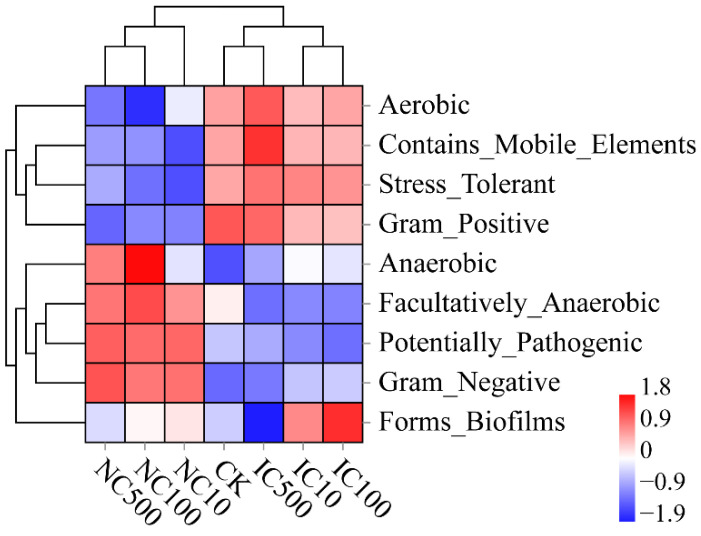
Hierarchical clustering heatmap of predicted bacterial phenotypic traits by BugBase. The heatmap is based on the standardized phenotypic trait abundance data. Warmer color (red) indicates a higher phenotypic trait abundance, while colder color indicates a lower phenotypic trait abundance.

The predicted phenotypic traits of the soil bacterial community are depicted in Figure 8. Consistently, NC treatments had a phenotypic patten dramatically different from that of IC treatments and CK. Aerobic taxa, those containing mobile elements, stress-tolerant taxa and Gram-positive taxa were less abundant in NC treatments than in IC and CK treatments. However, facultative anaerobes, potential pathogens and Gram-negative taxa were more abundant in NC treatments. Specially, the relative abundance of anaerobes in NC treatments was higher than in CK, with significant differences observed in NC100 and NC500 (*p* < 0.05).

## 4. Discussion

### 4.1. Effect Differentiations between Nanoceria and Ionic Cerium

In this study, cerium content in soil was found to be an important shaping factor of bacterial community structure (Figure 3B). After exposure to nanoceria, soil bacterial richness, diversity and evenness showed an increasing, but not significant, trend in all dose treatments compared to the control (CK) (Table S1). Smaller, and also non-significant, increases were also observed in the ionic cerium treatments compared with CK. Furthermore, the variations in soil bacterial community structure under cerium exposure were more obvious: the hierarchical bi-clustering heatmap, PCA biplot and CAP biplot collectively revealed that the effect of nanoceria on soil bacterial community was greater than that of ionic cerium (Figure 2 and Figure 3), as NC treatments were in one cluster, differentiated from IC treatments which were close to CK. Consistently with this, the same clustering pattern was also observed in the predicted bacterial functions and phenotypic traits. Generally, it implied that both nanoceria and ionic cerium can promote soil bacterial diversity, and nanoceria with all doses showed a greater promotion than ionic cerium did. The mechanism for this difference is suggested to be as follows: Ce is in a more bioavailable form as nanoceria due to improved transport to the bacteria surface compared with ionic cerium. Nanoforms as delivery agents are well known [9], but this hypothesis needs testing.

Few previous studies have focused on the microbial toxicity comparation of nanoceria and ionic cerium, especially regarding community diversity. Dahle and Arai [42] studied the effects of nanoceria and ionic cerium on soil denitrification and found that both nanoceria and ionic cerium at all doses exhibited significant inhibitory effects on depletion time and denitrification rate, and ionic cerium was far more toxic than nanoceria with an equal total cerium concentration, which seemed to be opposite to our promoting effect on bacterial diversity. However, those studies focused on the function of denitrifying microorganisms only, while our study paid attention to general community composition and the diversity of bacteria. For certain bacterial taxa, inhibitory effects on abundance were also observed in this study (Table 2), in agreement with Dahle and Arai [42].

The CAP revealed that TP, AP and AK were important factors correlated with the differentiation of IC and NC treatments other than residual Ce (Figure 3B), which indicated phosphorus and potassium to be key elements in driving the interactions between cerium and the bacterial community. Nanoceria was reported to be a promising phosphorus sorbent by the rapid forming of cerium–phosphate complexes [43,44]. It was predicted that soil microorganisms dissolve insoluble secondary phosphates by the production of organic ligands that compound with lanthanide ions including cerium [45]. Cervini-Silva et al. [46] found that rhabdophane dissolution is controlled either by strong ligand complexation of Ce^3+^(aq) or by sequestration of Ce^4+^ ions as CeO_2_(s), which effectively increases the mineral solubility. The interactions between organics, CePO_4_·H_2_O and CeO_2_(s) revealed in their study implied that there are important linkages among the cerium, phosphorus and organic carbon cycles in soil. Above all, it could be predicted that nanoceria and ionic cerium might directly affect phosphorus availability by the formation of cerium–phosphate complexes, which could account for the significant increase in total phosphorus and significant decrease in available phosphorus in all cerium treatments (Table 1). Consequently, the variation in available phosphorus further influenced the soil bacterial community structure [47]. It has been reported that potassium can act as a modifier or chemical promoter to improve the performance and stability of CeO_2_ as a catalyst [48]. However, the detailed interaction mechanisms among soil bacterial, nutrients (P and K) and nanoceria or ionic cerium still need further verification.

### 4.2. Bacterial Co-Occurrence Patterns under Exposure to Cerium

The co-occurrence networks showed that the IC treatments had a more complex network than nanoceria treatments according to their topological properties. This indicated that the connections between bacterial taxa were weaker after nanoceria exposure than ionic cerium exposure. This implied that soil bacteria were responsive in community structure under cerium exposure. To be detailed, in the networks, the top ten keystone genera revealed by the betweenness centrality score of each node were entirely different except for two shared genera, namely *Steroidobacter* and *Acidovorax*. Hamidat et al. [13] found that nanoceria treatment resulted in an increased relative abundance of *Acidovorax*, a degrader of polycyclic aromatic hydrocarbons (PAHs). They assumed that *Acidovorax* potentially harbors tolerance to heavy metals as well as resistance to antibiotics and multi-drugs just as other hydrocarbon-degrading bacteria do [49,50,51]. Therefore, the presence of *Acidovorax* in the top ten keystone taxa in both the NC and IC networks in this study could also be attributed to its tolerance or resistance to cerium.

For the keystone taxa in the NC network, previous studies found that the relative abundance of *Bdellovibrio* and *Terrimonas* increased under exposure to nanoceria [52,53], while the relative abundance of *Arenimonas* decreased [53]. For the keystone taxa in the IC network, we speculate that the role of *Aeromonas* might be attributed to its potential ability to synthesize nanoparticles using ionic cerium as a substrate; it was reported to be capable of synthesizing several kinds of nanoparticles previously [54,55,56]. *Acidovorax* was found to be markedly resistant to Ag^+^ rather than nanosilver [57], based on which it could be preliminarily assumed that *Acidovorax* would also be resistant to Ce^3+^ rather than nanoceria, although this needs testing. That is why *Acidovorax* was a top keystone taxon in the IC network but not in the NC network. Other keystone taxa in both networks were not referred to in previous studies, but it could be predicted that they might be responsive, tolerant or resistant to nanoceria or ionic cerium so they were stimulated under corresponding exposure.

### 4.3. Responsive Bacterial Taxa, Functions and Phenotypes

The composition, activity and biomass of soil microbial communities are sensitive indicators of soil response to environmental stress. In this study, the two-way ANOVA revealed that both Ce species and dose played significant roles in affecting the abundance of bacterial taxa at the phylum level, especially the dominant ones (Table S3). However, no significant interactive effects of Ce species and dose were found, which indicated that Ce species and dose solely affected soil bacterial community composition. Among the top ten phyla, the abundance of Acidobacteria, Actinobacteria, Verrucomicrobia, Chloroflexi and Gemmatimonadetes was significantly affected by both Ce species and dose. Proteobacteria and Bacteroidetes were just significantly affected by Ce species. To be more detailed, the bacterial biomarkers were screened out (Figure 4).

Under exposure to nanoceria, Proteobacteria (Nitrosomonadaceae, *Aeromonas*, Pseudomonadaceae, SZB85 and MND1), Acidobacteria (Subgroup_6 and *Stenotrophobacter*), Rokubacteria (c_NC10 and Rokubacteriales), Planctomycetes (WD2101_soil_group) and Gemmatimonadetes (Gemmatimonadaceae) were significantly enriched. Among them, Proteobacteria and Rokubacteria showed a positive dose-dependent correlation with nanoceria (Table 2). More interestingly, Nitrosomonadaceae, MND1 (Nitrosomonadaceae), SZB85 (Nitrosococcaceae), Nitrospiraceae and Rokubacteria could function as a comammox, anammox and nitrifier [58,59,60,61]. Thus, it could generally be summarized that Proteobacteria and nitrifying bacteria are the most responsive taxa to nanoceria exposure.

Hamidat et al. [13] also found that the abundance of Proteobacteria in canola plants significantly increased from nanoceria treatment in comparison to the control. However, Kamika and Tekere [62] reported that the abundance of Proteobacteria decreased as the concentration of nanoceria increased in activated sludge. Miroshnikov et al. [63] claimed that one of the peculiar actions of nanoceria on zebrafish’s intestinal microbiota was the decrease in Proteobacteria phylum abundance. The differing effects of nanoceria on the abundance of Proteobacteria could be related to the different systems or to the different physicochemical properties of the NPs (size, Ce^3+^/Ce^4+^ ratio, O2 storage and enzymatic-mimetic activities of the nanoparticle [64]), and this needs further investigation. The particle diameter (crystallite size) of the nanoceria used by Hamidat et al. [13] was 31 nm, which was similar to that of our study (25 nm). However, the particle size was much smaller (15.8 nm) in Miroshnikov et al. [63]. The nanoceria diameter was not stated in Kamika and Tekere [62]. It was previously assumed that toxicity increases as the nanoparticle size becomes smaller, because smaller particles harbor a larger surface area per mass unit and are thus potentially more reactive [65,66,67]. For nanoceria, a higher specific surface area leads to a larger surface Ce^3+^/Ce^4+^ ratio, which could contribute to a higher toxicity for smaller nanoceria [68,69].

In addition, the different effects of nanoceria on Proteobacteria could also be due to the different exposure scenarios. Most environmental exposure scenarios of nanoparticles were in aqueous conditions and in media with different chemical and biological properties. Nanoceria acts as a colloid in aqueous body fluids and soil environments, and its surface chemistry, dispersity, reactivity and mobility can be changed by the adsorbed materials and environmental factors such as temperature, surface atomic arrangements, ions in solution, pH, and inorganic or organic ligands adsorbed on its surface [70]. Thus, the environmental and biological effects of nanoceria were changed and varied in different exposure scenarios [43]. The exposure environments of Hamidat et al. [13] and this study were both planting soils. Edaphic physicochemical properties such as texture, porosity, pH, ionic strength, organic matter and mineral composition play a critical role in determining the aggregation, dissolution, sorption, chemical transformation, bioavailability, reprecipitation and migration of nanoparticles in media [71]. These would lead to variations in the effects of nanoparticles on soil microbial communities. For example, the presence of natural organic matter and ionic strength determine the electrophoretic mobility, transport characteristics and toxicity of nanoparticles in the environment [72]. Therefore, greater differences would be observed when the media are different, such as those in the previous referenced studies including planting soil, activated sludge and zebrafish’s intestines [13,62,63]. To date, no model can quantify this complexity. Moreover, Proteobacteria is a bacterial phylum including a large number of taxa with different physiological and functional characteristics. To some extent, the identified increase or decrease in Proteobacteria abundance was an overall superimposed effect of all the specific taxa’s responses after cerium exposure. The differences in detailed taxonomic composition and predominance within Proteobacteria between different studies also contribute to the consistence or inconsistence. Therefore, future studies should try to elucidate mechanisms by investigating these exposure changes in controlled ways.

On the other hand, the identification of nitrifying bacteria, including Nitrosomonadaceae, MND1 (Nitrosomonadaceae), SZB85 (Nitrosococcaceae), Nitrospiraceae and Rokubacteria, as biomarkers of nanoceria exposure (Figure 4A) was also found in a previous study [73]. Just like Proteobacteria, nitrifiers were also mostly found to be damaged by the cytotoxicity of nanoceria [74,75,76], inconsistent with our study. However, Yu et al. [75] also reported that there was a possible establishment of an anti-toxicity mechanism in *Nitrosomonas europaea* under nanoceria exposure. This anti-toxicity mechanism may be ubiquitously established in nitrifying bacteria. Of course, the inconsistent effects of nanoceria among different studies could also be due to the differences in those nanoparticle properties and exposure scenarios as discussed above for Proteobacteria.

Under exposure to ionic cerium, Planctomycetes (Phycisphaerae, Tepidisphaerales and Gemmataceae) were the main biomarkers for ionic cerium disturbance. This might be because Planctomycetes were a group of methylotrophic or anammox bacteria, and ionic cerium acted as a cofactor for methanol dehydrogenase in methylotroph [77].

Although the significant variation in most of those bacterial biomarkers was not dose-dependent, it still indicated that the bacterial community was responsive under either nanoceria or ionic cerium exposure stress, which was in accordance with the indicator role of microorganisms for soil perturbation or xenobiotic pollutants (Tai et al., 2020).

In our study, the differentiation of NC from CK and IC was not only observed in the community composition but also in the predicted functions and predicted phenotypes (Figure 7 and Figure 8). The most remarkable phenomenon would be the enrichment of anaerobic and facultatively anaerobic phenotypes in NC, together with the reduction in the aerobic phenotype. It might contribute to the protecting effect of nanoceria as it could adhere to the cellular surface, suppress the production of reactive oxygen species and induce cellular resistance to an exogenous source of oxidative stress [78]. This facilitates the anaerobes to be more tolerant to oxidative stress from the environment. Furthermore, it is also noticeable that nanoceria seemed to promote Gram-negative phenotypes and inhibit Gram-positive ones, which still needs further investigation to confirm in the future.

Collectively, our study systematically revealed the different response patterns of soil bacteria to exposure to nanoceria and ionic cerium from several perspectives including community diversity, biomarkers, predicted functions and phenotypes and co-occurrence. We believe it will help to unveil the previous uncertainties about the microbial ecological effects of nanoceria. However, the detailed mechanisms of these effects still need further investigation. Therefore, future work should comprise more concrete studies to reveal the microbial biomarker effects of nanoceria and ionic cerium at the genomic, transcriptomic and metabonomic levels.

## 5. Conclusions

Our study demonstrated that nanoceria and ionic cerium both promote soil bacterial diversity. The bacterial composition, predicted functions and phenotypes under nanoceria exposure were dramatically different from those under ionic cerium exposure and had a less complex co-occurrence network. Proteobacteria and nitrifying bacteria were the most responsive biomarkers to nanoceria exposure. The biomarkers under ionic cerium exposure were mainly taxa of Planctomycetes. Anaerobic and Gram-negative bacteria were enriched under nanoceria exposure. Soil cerium together with potassium and phosphorus were key factors driving the differentiation.

## Figures and Tables

**Table 1:** Soil physicochemical properties in different treatments.

	SOM g/kg	TN g/kg	AN mg/kg	TP g/kg	AP mg/kg	TK g/kg	AK mg/kg	Ce mg/kg
NC10	17.22 ± 1.27 ^a^	0.19 ± 0.02 ^a^	75.21 ± 3.01 ^a,b^	0.78 ± 0.03 ^a^	18.51 ± 2.69 ^c^	19.02 ± 0.51 ^b^	107.19 ± 3.01 ^b,c^	10.09 ± 2.63 ^d^
NC100	17.22 ± 1.06 ^a^	0.16 ± 0.01 ^a^	55.97 ± 3.19 ^b^	0.7 ± 0.06 ^a^	22.68 ± 1.24 ^c^	19.19 ± 0.67 ^b^	119.32 ± 15.47 ^b,c^	38.87 ± 0.66 ^c^
NC500	17.14 ± 0.98 ^a^	0.18 ± 0.001 ^a^	73.16 ± 10.01 ^a,b^	0.78 ± 0.01 ^a^	23.43 ± 2.63 ^c^	19.09 ± 0.21 ^b^	107.53 ± 25.43 ^bc^	135.03 ± 28.91 ^a^
IC10	16.33 ± 1.04 ^a^	0.16 ± 0.003 ^a^	56.78 ± 2.51 ^b^	0.65 ± 0.07 ^a,b^	43.3 ± 7.28 ^a,b^	20.34 ± 0.96 ^b^	141.71 ± 7.96 ^b^	9.57 ± 2.56 ^d^
IC100	17.26 ± 0.65 ^a^	0.17 ± 0.01 ^a^	58.01 ± 6.07 ^b^	0.64 ± 0.005 ^a,b^	32.45 ± 3.64 ^b,c^	18.89 ± 0.68 ^b^	124.21 ± 3.96 ^bc^	44.43 ± 0.65 ^c^
IC500	17.99 ± 1.1 ^a^	0.19 ± 0.02 ^a^	80.81 ± 20.1 ^a,b^	0.74 ± 0.003 ^a^	27.85 ± 0.63 ^c^	19.8 ± 0.58 ^b^	95.09 ± 3.88 ^c^	103.13 ± 14.10 ^b^
CK	17.19 ± 0.9 ^a^	0.21 ± 0.02 ^a^	101.97 ± 2.66 ^a^	0.51 ± 0.08 ^b^	49.63 ± 6.68 ^a^	22.39 ± 0.48 ^a^	252.36 ± 16.7 ^a^	9.74 ± 1.24 ^d^

Notes: NC = nanoceria; IC = ionic cerium; the number means the concentration of cerium (mg/kg); the same below. SOM = soil organic matter; TN = total nitrogen; AN = available nitrogen; TP = total phosphorus; AP = available phosphorus; TK = total potassium; AK = available potassium; Ce = soil residual cerium concentration. Different superscript lowercase letters within each column mean significant differences (*p* < 0.05) between different treatments revealed by one-way ANOVA.

**Table 2:** Bacterial taxa significantly correlated with cerium dose at the phylum level.

Cerium Type	Bacterial Taxa	*r*	*p*
Nanoceria	Proteobacteria	0.67	0.017
	Lentisphaerae	0.62	0.033
	Rokubacteria	0.60	0.037
	Epsilonbacteraeota	−0.60	0.037
Ionic cerium	Hydrogenedentes	0.59	0.043
	Bacteroidetes	−0.71	0.009

**Table 3:** Topological properties of bacterial co-occurrence networks under different treatments.

	Node	Edge	Average Degree	Network Diameter	Graph Density	Modularity	Clustering Coefficient	Average Path Length
NC	82	101	2.463	9	0.03	0.781	0.534	3.632
IC	91	214	4.703	16	0.052	0.719	0.515	5.233

## Data Availability

Not applicable.

## Supplementary Materials

The following supporting information can be downloaded at: https://www.mdpi.com/article/10.3390/microorganisms10101982/s1, Figure S1: SEM micrograph of nanoceria particles used in this study (acceleration voltage of 5.0 kV, magnification of 170,000×); Figure S2: Rarefaction curves of bacterial 16S rRNA gene sequencing in different treatments; Figure S3: Network of co-occurring bacterial genera of nanoceria (A) and ionic cerium (B) treatments based on Spearman correlation analysis, sorted in color by modularity; Table S1: Alpha diversity indexes among different treatments; Table S2: Summary of the taxonomic classification of all samples; Table S3: Two-way ANOVA of cerium’s effect on soil bacterial phyla abundance.
