# Supplementary material for: Response of Soil Bacterial Diversity, Predicted Functions and Co-Occurrence Patterns to Nanoceria and Ionic Cerium Exposure
Source: Microorganisms. 2022 Oct 6;10(10):1982. doi: 10.3390/microorganisms10101982 (PMC9607988; doi:10.3390/microorganisms10101982)
Supplement: Supplementary file 1 [file microorganisms-10-01982-s001.zip › microorganisms-1929140-supplementary.pdf]

Table S1. Alpha diversity indexes among different treatments.

	Sobs Richness	Shannon–Weaver Diversity	Pielou Evenness	Goods Coverage
NC10	4038±382[ab]	9.75±0.28[a]	0.8145±0.01[a]	0.9907±0.0003[ab]
NC100	4067±160[ab]	9.75±0.04[a]	0.8136±0.003[a]	0.9902±0.0006[ab]
NC500	4098±158[ab]	9.77±0.15[a]	0.8140±0.01[a]	0.9900±0.0004[bc]
IC10	3932±138[ab]	9.68±0.09[a]	0.8106±0.01[a]	0.9903±0.0007[ab]
IC100	3837±96[b]	9.63±0.07[a]	0.8186±0.003[a]	0.9903±0.0003[ab]
IC500	4284±191[a]	9.69±0.20[a]	0.8035±0.02[a]	0.9888±0.0016[c]
CK	3766±65[b]	9.64±0.03[a]	0.8117±0.002[a]	0.9915±0.0002[a]

NC = nanoceria; IC = ionic cerium; the number means the concentration of cerium (mg/kg); CK = negative control.

Table S2. Summary of the taxonomic classification of all samples.

Sample ID	Number of Taxa					
	Phylum	Class	Order	Family	Genus	OTUs
NC10-1	30	92	167	247	392	4513
NC10-2	28	84	159	220	347	3742
NC10-3	30	90	169	232	352	3914
NC100-1	30	92	163	236	360	3859
NC100-2	29	93	176	241	392	4176
NC100-3	28	89	166	225	383	4141
NC500-1	30	91	162	226	344	3913
NC500-2	30	92	172	240	426	4181
NC500-3	28	90	172	229	358	4196
IC10-1	29	86	161	227	373	4096
IC10-2	29	85	159	221	346	3857
IC10-3	29	88	158	222	369	3886
IC100-1	25	83	151	208	329	3754
IC100-2	30	87	157	225	354	3854
IC100-3	25	77	153	225	365	3985
IC500-1	29	90	167	235	372	4094
IC500-2	31	93	183	254	446	4484
IC500-3	30	90	172	244	387	4244
CK-1	30	88	164	231	381	3826
CK-2	29	83	151	227	381	3702
CK-3	26	82	154	225	366	3771
All	35	108	222	334	661	8874

Table S3. Two-way ANOVA of cerium's effect on soil bacterial phyla abundance.

Carbon Sources	Ce Species	Ce Dose	Interaction
Planctomycetes	NS	NS	NS
Proteobacteria	*	NS	NS
Acidobacteria	**	*	NS
Actinobacteria	*	*	NS
Verrucomicrobia	*	*	NS
Chloroflexi	*	*	NS
Firmicutes	NS	NS	NS
Patescibacteria	NS	NS	NS
Gemmatimonadetes	*	*	NS
Bacteroidetes	*	NS	NS
BRC1	*	NS	·
Rokubacteria	*	NS	NS
Cyanobacteria	NS	*	NS
Armatimonadetes	*	**	NS
Nitrospirae	**	**	·
Deinococcus-Thermus	NS	NS	NS
Tenericutes	NS	NS	NS
Hydrogenedentes	NS	**	NS
Epsilonbacteraeota	NS	**	NS
Elusimicrobia	*	NS	NS
Chlamydiae	NS	NS	·
Spirochaetes	NS	NS	NS
Fibrobacteres	*	NS	**
Omnitrophicaeota	·	NS	NS
Kiritimatiellaeota	NS	NS	NS
WPS-2	·	NS	NS
Dependentiae	NS	NS	NS
Deferribacteres	NS	·	NS
Dadabacteria	NS	NS	NS
Lentisphaerae	NS	NS	NS
Latescibacteria	NS	NS	NS

·, $P < 0.1$; *, $P < 0.05$; **, $P < 0.01$.

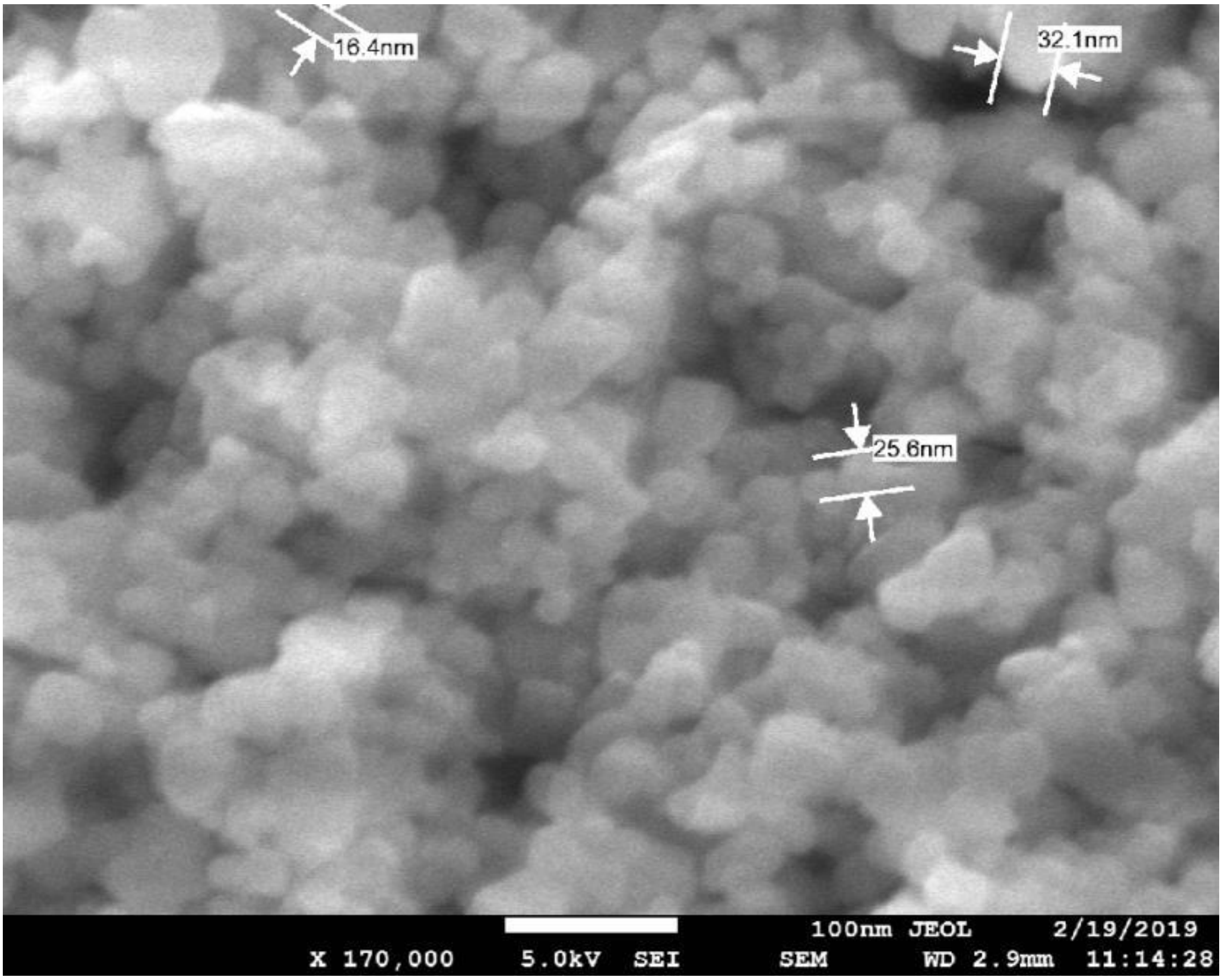

Figure S1. SEM micrograph of nanoceria particles used in this study (acceleration voltage of 5.0 kV, magnification of 170,000×).

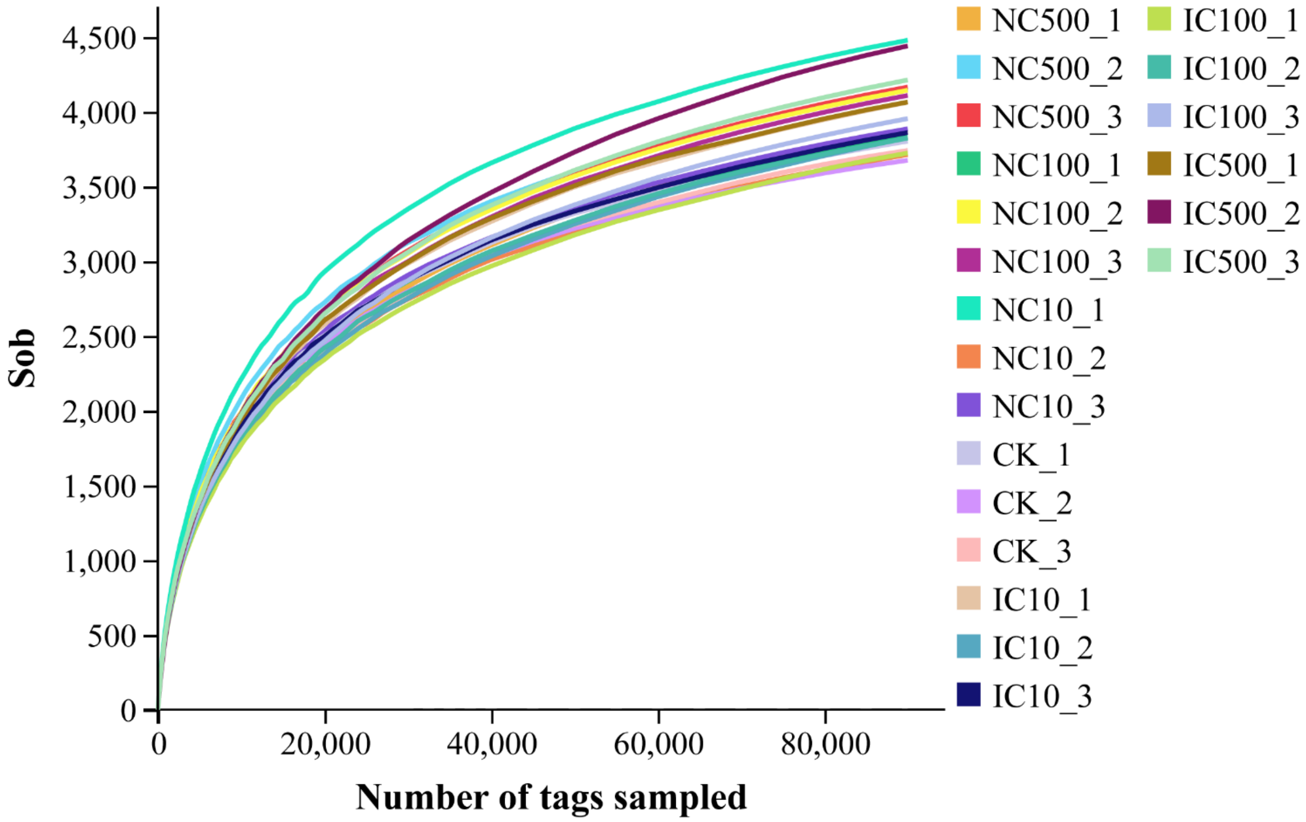

Figure S2. Rarefaction curves of bacterial 16S rRNA gene sequencing in different treatments.

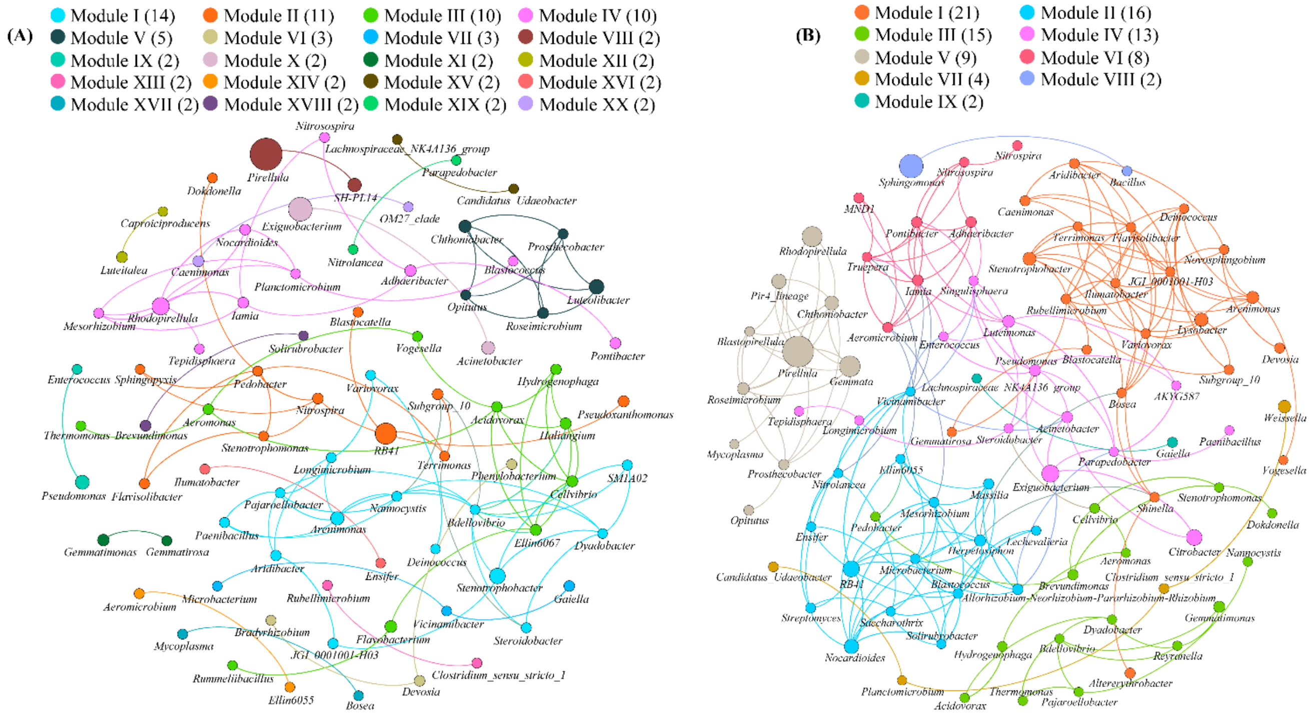

Figure S3. Network of co-occurring bacterial genera of nanoceria (A) and ionic cerium (B) treatments based on Spearman correlation analysis, sorted in color by modularity. A connection indicates a significant (*r*>0.6, *P*<0.01) correlation. The size of each node (genus) is proportional to its abundance; the thickness of each connection between two nodes (edge) is proportional to the corresponding correlation coefficient.
